# Parental perception of child's weight, their attitudes towards child's dietary habits and the risk of obesity

**DOI:** 10.1007/s12519-022-00540-6

**Published:** 2022-03-25

**Authors:** Lorena García-Blanco, Arantxa Berasaluce, Andrea Romanos-Nanclares, Miguel Ángel Martínez-González, Laura Moreno-Galarraga, Nerea Martín-Calvo

**Affiliations:** 1 San Juan Primary Care Health Center, Servicio Navarro de Salud-Osasunbidea, Pamplona, Spain; 2 IdiSNA, Instituto de Investigación Sanitaria de Navarra, Pamplona, Spain; 3 Department of Preventive Medicine and Public Health, School of Medicine, University of Navarra, C/ Irunlarrea 1, 31080 Pamplona, Spain; 4 Biomedical Research Centre Network On Obesity and Nutrition (CIBERobn), Physiopathology of Obesity and Nutrition, Institute of Health Carlos III, Av. Monforte de Lemos, 3-5, 28029 Madrid, Spain; 5 Department of Nutrition, Harvard T.H. Chan School of Public Health, Boston, MA 02115 USA; 6 Department of Pediatrics, Complejo Hospitalario de Navarra B, Pamplona, Spain

**Keywords:** Childhood obesity, Dietary attitudes, Eating habits, Overweight, Weight perception

## Abstract

**Background:**

The association between parental perception of child’s weight and their attitudes towards his/her dietary habits has not been reported yet. This study aimed to assess the association between parental underestimation of child’s weight and parental attitudes towards child’s dietary habits.

**Methods:**

We conducted a cross-sectional analysis of SENDO cohort participants recruited between January 2015 and June 2020. All information was collected through online questionnaires completed by parents. We calculated crude and multivariable-adjusted odds ratio (OR) and 95% confidence intervals (CI) for unhealthy attitudes towards child’s dietary habits associated with parental underestimation of child’s weight.

**Results:**

Sixteen percent of children in the SENDO project had parents who underestimated their weight. Parents who underestimated their child’s weight status were more likely to have unhealthy attitudes toward his/her dietary habits [OR 3.35; 95% CI (1.71–6.53)].

**Conclusions:**

Parental underestimation of child's weight was associated with unhealthy attitudes towards child’s dietary habits. Pediatricians and public health practitioners should pay attention to the parental perception of child’s weight to identify parents who underestimate it as an at-risk group in which to inquire about lifestyle and dietary habits.

**Supplementary Information:**

The online version contains supplementary material available at 10.1007/s12519-022-00540-6.

## Introduction

Perception is the construction of mental symbols or representations of reality acquired through the senses [1], and because it can be influenced by various factors, perception is not always faithful to reality. Child’s age, sex and family history of obesity are the factors most often found to be associated with a parental misperception of child’s weight [2–7]. Child’s body mass index (BMI) deserves a special mention. Most of the evidence on the relationship between parental perception of weight and actual child’s BMI *z* scores comes from cross-sectional studies [5, 6]; therefore, the direction of that association and whether it represents a true biological effect remain unclear.

The high prevalence of parental underestimation of child’s weight status [4] has important public health implications. Evidence on the association of weight misperception with lifestyle [3, 8, 9] and feeding practices [10, 11] is scarce and inconsistent; therefore, the extent to which misperception represents an obstacle to obesity prevention is unknown [12].

Attitudes are emotional, motivational, perceptive, and cognitive beliefs that influence the behavior or practice of an individual regardless of whether he/she has knowledge [13]. Far from being static and fixed, attitudes are continuously shaped by intrinsic (genetic, age, and sex) and environmental factors, such as family, friends, or neighborhood [14]. Parents are important agents in the promotion of the health of their children. Parents play a key role in structuring their children’s first experiences with food and eating through their own beliefs, food practices, perspectives, eating attitudes, dietary knowledge, and understanding of the benefits of food and nutrients on health [15]. Parental nutrition knowledge and dietary attitudes have been described as particularly important factors for children’s healthy food knowledge [15].

To our knowledge, the association between parental perception of child’s weight and parental attitudes towards child’s dietary habits has not been studied. In this scenario, we aimed to assess the association between parental underestimation of child’s weight and parental attitudes towards child’s dietary habits using data from the SENDO project (from Spanish Child’s follow-up for an optimal growth), an ongoing cohort of Spanish children.

## Methods

### Study population

The SENDO project is a prospective pediatric cohort focused on studying the effect of diet and lifestyle on children’s and adolescents’ health. The SENDO project started in 2015 with a small sample of children from Pamplona (Spain). Since 2017, the recruitment has been permanently open to new participants country wide. Potential participants are contacted at their primary care center or at school. Additionally, any potential participant can request entry into the cohort using the project’s website (www.proyectosendo.es). Inclusion criteria are: (1) children between 4 and 5 years old at recruitment date, and (2) who are living in Spain. All participants’ parents signed an informed consent at recruitment. Exclusion criteria are the lack of signed informed consent or an electronic device with internet access.

Information is collected at baseline and is updated every year through online, self-administered questionnaires completed by parents. For the present study, we used baseline information of participants recruited between January 2015 and June 2020. The baseline questionnaire includes 4 sub-questionnaires in the following areas: (1) sociodemographic data, (2) eating habits, (3) dietary intake, through a validated semi-quantitative food frequency questionnaire [16] (FFQ), and (4) physical activity.

Of the 734 participants recruited before June 2020, 51 had not completed the baseline questionnaire at the time this study started, and they were excluded. In addition, 9 participants were excluded owing to missing data on parental perception of child’s weight status. Therefore, the final sample for this study included 674 children with complete information.

### Exposure assessment

Parental perception of children’s weight was collected on a verbal scale with five categories of response (severely underweight, underweight, normal weight, overweight and obese). The two higher categories and the two lower categories were merged owing to the small number of responses; therefore, parental perception of child’s weight status was categorized as low, normal or high. Underestimation was defined as parental perception lower than the actual child’s weight status as defined by the *z* score of the BMI. In the main statistical analyses, correct estimation was used as the reference category.

### Ascertainment of the outcome

Parental attitudes toward their child’s dietary habits were evaluated using 8 yes/no items related to parental predisposition to certain habits regarding their child’s diet (i.e., “I try my child to eat more fruit”). Affirmative answers were assigned 1 point, and negative answers were assigned 0 points. Hence, the final scored ranged from 0 to 8 points, with a higher score suggesting healthier attitudes. For descriptive purposes, participants were classified as having unhealthy (0–3 points), medium (4–5 points) or healthy attitudes (6–8 points). For the main statistical analyses, medium and healthy categories were merged to yield only two categories: healthy (≥ 4 points) or unhealthy (< 4 points) attitudes towards child’s dietary habits.

### Assessment of covariates

The questionnaire collected information on sociodemographic variables, family and personal medical history, food frequency intake, dietary habits and lifestyle. Both child’s and mother’s ages were calculated as the difference between the date on which the questionnaire was received and their respective birth dates.

BMI was calculated as the ratio between reported weight (kg) and height-squared (m^2^). Information about the standard procedures to collect these data was indicated in the questionnaire. Nutritional status was defined using sex- and age-specific cut-off points of the BMI based on the International Obesity Task Force (IOTF) standards of reference [17]. Age- and sex-specific z-score of the BMI was calculated using the LMS method [17].

A trained dietitian derived the nutrient content of each item in the FFQ [16] using updated Spanish food composition tables[18] and online databases [19, 20]. Nutrient content of each food item was calculated by multiplying the intake frequency by the edible portion and by the nutrient composition of the specified portion size. Total energy intake was obtained by summing up the contribution of each item.

Physical activity was collected with a questionnaire that included 17 activities and 10 categories of response, from never to 11 or more hours/week. Metabolic Equivalent of Task (METs)-h/week for each activity were calculated by multiplying the number of METs of each activity by the weekly participation in that activity, weighted according to the number of months dedicated to each activity [21]. Total physical activity was quantified by summing the METs-h/week dedicated to all activities performed during leisure time.

Screen time was calculated as the mean of hours/day spent watching television, using a computer or playing video games during weekdays and weekends. Screen time was used as a proxy for sedentary behavior [22].

Parental knowledge on nutritional recommendations for children was evaluated with questions about recommended intake frequency of 18 different food groups. The nine categories of response ranged from “Never” to “6 or more times per day”. Each question was assigned one point if the answer met the dietary recommendations and 0 points if it did not [23]. The final score was expressed as a percentage, with a higher value meaning higher knowledge on nutritional recommendations for children. For the statistical analyses, participants were categorized as having high (> 70%), medium (40–70%) or little (< 40%) dietary knowledge. Little knowledge was used as the reference category.

Diet quality was defined using the KIDMED score, an a priori defined dietary index that evaluates the adherence to the Mediterranean dietary pattern in children and adolescents. The KIDMED index consists of 16 items, 12 items were scored as 0 or + 1, whereas 4 items were scored as -1 or 0. Thus, the KIDMED score ranges from − 4 to 12 points [24]. According to their score, participants were categorized as having a poor (≤ 3 points), medium (4–7 points), or high adherence (≥ 8 points) to the Mediterranean dietary pattern [25, 26].

### Statistical analysis

We described the participant’s characteristics by parental perception (underestimation, agreement or overestimation) of child’s weight. The results were presented as numbers (percentages) for categorical variables and as means (standard deviations) for quantitative variables. We compared underestimation vs. agreement and overestimation vs. agreement using Student’s *t* test for quantitative variables and using chi-squared test for qualitative variables.

In the main analyses, we calculated crude and multivariable-adjusted odds ratios (OR) and their 95% confidence interval (CI) for unhealthy attitudes towards child’s dietary habits associated with parental underestimation compared to correct estimation of their child’s weight. Odds ratios were estimated using a multilevel, mixed-effects generalized linear model of binomial counts. This model was specified using the following options: family (binomial), link (logit) and vce(cluster), which account for correlation among siblings when estimating standard errors. Multivariable analyses were progressively adjusted for (1) sex and race (white or others); (2) KIDMED score (low, medium or high), screen time (continuous) and frequency of television watching during meals (≤ 3 times/month, 1–3 times/week, ≥ 4 times/week); and (3) *z* score of the BMI (continuous).

Analyses were carried out using Stata version 15.0 (Stata Corporation). All *P*-values were two-tailed. Statistical significance was assigned using the conventional cut-off point of *P* < 0.05.

### Ethical standards

The SENDO project follows the rules of the Declaration of Helsinki on the ethical principles for medical research in human beings. This study was approved by the Ethics Committee removed for blind peer review. An informed consent was obtained from all participants’ parents at recruitment.

## Results

At recruitment, children's mean (SD) age was 5.1 (0.8) years, and 347 (51%) of the children were boys. Of the 674 participants (including 596 clusters, i.e., different families) who had completed the baseline questionnaire when this study started, 111 (16%) had parents who underestimated their weight and 67 (10%) had parents who overestimated it.

Participant’s main characteristics by the parental perception of child’s weight are presented in Table 1. Mothers were the person who most often completed the questionnaire in all 3 groups. No differences were observed between groups for maternal age, the percentage of mothers with higher education, family history of obesity or the number of children. Parents in the three groups reported similar knowledge on nutritional recommendations for children. Regarding children’s characteristics, no differences were observed for age and sex distribution, but regarding race, those who underestimated it were less often white (91.89% vs. 97.38%; *P* = 0.005).

**Table 1:** Characteristics of participants in the SENDO project by parental estimation of their offspring weight status

Variables	Underestimation	Correct estimation	Overestimation
	*n* = 111	*n* = 496	*n* = 67
*Parental characteristics*			
Questionnaire responder, %			
Only the mother	83 (74.77)	401 (80.85)	55 (82.09)
Only the father	14 (12.61)	33 (6.65)	7 (10.45)
Both mother and father	14 (12.61)	58 (11.69)	5 (7.46)
Maternal age (y)	39.80 (4.17)	40.08 (3.97)	39.77 (3.58)
Maternal high education, %	88 (79.28)	408 (82.26)	55 (82.09)
Family history of obesity, %	18 (16.22)	98 (19.76)	13 (19.40)
Knowledge about child’s nutritional recommendations, %			
Low score (< 40%)	23 (20.72)	117 (23.59)	12 (17.91)
Medium score (40–70%)	73 (65.77)	312 (62.90)	46 (68.66)
High score (> 70%)	15 (13.51)	67 (13.51)	9 (13.43)
Number of children, %			
1–2	63 (56.76)	287 (57.86)	47 (70.15)
3–4	25 (22.52)	121 (24.40)	11 (16.42)
5 or more	23 (20.72)	88 (17.74)	9 (13.43)
*Children characteristics*			
Sex (female), %	52 (46.85)	241 (48.59)	34 (50.75)
Age (y)	5.13 (0.83)	5.07 (0.89)	4.97 (0.88)
Race (white), %	102 (91.89)	483 (97.38)	66 (98.51)
Gestational age, %			
< 38 wk	19 (17.27)	71 (14.37)	9 (13.64)
38–40 wk	45 (40.91)	192 (38.87)	35 (53.03)
> 40 wk	46 (41.82)	231 (46.76)	22 (33.33)
Birthweight (g)	3228 (653)	3247 (526)	3135 (541)
Breastfeeding duration, %			
No breastfeeding	22 (19.82)	76 (15.32)	7 (10.45)
< 3 mon	11 (9.91)	63 (12.70)	6 (8.96)
3–6 mon	25 (22.52)	108 (21.77)	5 (7.46)
> 6 mon	53 (47.75)	249 (50.20)	49 (73.13)
Position among siblings, %			
The oldest/singletons	38 (34.23)	189 (38.10)	27 (40.30)
2 nd/3 or 2 nd-3 rd/4	20 (18.02)	100 (20.16)	10 (14.93)
The youngest or beyond the 4th	53 (47.75)	207 (41.73)	30 (44.78)
Z-score of the BMI	0.81 (1.23)	0.10 (0.88)	-1.16 (0.96)
Kidmed score			
Low (≤ 3 points)	14 (12.61)	38 (7.66)	7 (10.45)
Medium (4–7 points)	77 (69.37)	349 (70.36)	52 (77.61)
High (≥ 8 points)	20 (18.02)	109 (21.98)	8 (11.94)
Total energy intake (kcal/d)	2091 (492)	2081 (502)	2035 (575)
Physical activity (METs-h/wk)	38.96 (27.98)	42.02 (31.06)	32.54 (19.69)
Screen time (hours/day)	1.42 (0.89)	1.24 (0.94)	1.00 (0.74)
Snacking, %			
≤ 3 times/mon	58 (53.21)	292 (60.33)	42 (64.62)
1–3 times/wk	39 (35.78)	141 (29.13)	18 (27.69)
≥ 4 times/wk	12 (11.01)	51 (10.54)	5 (7.69)
TV watching during meals, %			
≤ 3 times/mon	63 (56.76)	312 (62.90)	36 (53.73)
1–3 times/wk	17 (15.32)	97 (19.56)	18 (26.87)
≥ 4 times/wk	31 (27.93)	87 (17.54)	13 (19.40)

Numbers are mean (SD) or *N* (%)

Parental underestimation of child’s weight was not associated with gestational age, children’s position within the family or breastfeeding history. However, the *z* score of the BMI was significantly higher in the group of children whose parents underestimated their weight (diff. = 0.71; 95% CI [0.47–0.96]) and was lower in the group of children whose parents overestimated it (diff. = −  1.25 (− 1.50 to − 1.00) compared with the reference group (correct perception).

Regarding lifestyle variables, underestimation was not associated with energy intake, physical activity or snacking. A marginally significant difference (*P* = 0.19) was observed for adherence to the Mediterranean dietary pattern, with lower adherence in children whose parents underestimated their weight as compared with those who correctly perceived it. Children whose parents underestimated their weight were more likely to watch television during meals (*P* = 0.04) and spent more screen-time (*P* = 0.06). On the other hand, parents who overestimated their child’s weight reported a longer duration of the breastfeeding (*P* = 0.004), less physical activity (diff. = − 9.48, METs-h/week; 95% CI [-14.99 to -3.97]) and less screen-time (diff. = − 0.24 h/day; 95%CI [0.04–0.44]) than those who correctly perceived child’s weight.

The main analyses included 607 children (111 in the underestimation group and 496 in the correct estimation group) in 543 clusters (i.e., different families). After accounting for sex and race, parental underestimation of the child’s weight was associated with significantly higher odds of unhealthy attitudes towards child’s dietary habits [OR (95% CI): 3.15 (1.60–6.18)] (Table 2). Similar estimates were obtained when we progressively adjusted for lifestyle variables OR (95% CI) for unhealthy attitudes: 2.88 (1.48–5.60) and child’s *z* score of BMI [OR (95% CI)] for unhealthy attitudes: 3.35 (1.71–6.53) (Table 2). The estimates (95%) for the other factors analyzed in the multivariable adjusted are presented in Supplementary table1.

**Table2:** Odds ratio (OR) and 95% confidence interval (CI) for parental unhealthy attitudes towards child’s dietary habits associated with underestimation of child’s weight

	Correct estimation of child’s weight status	Underestimation of child’s weight status	
Number of participants	496	111	
Number of participants whose parents reported unhealthy attitudes towards child’s dietary habits	22	14	

	Odds ratio (95% CI)		*P*
Crude model	1 (Ref.)	3.10 (1.60–6.03)	< 0.005
Sex and race adjusted model	1 (Ref.)	3.15 (1.60–6.18)	< 0.005
Multivariable adjusted model^†^	1 (Ref.)	2.88 (1.48–5.60)	< 0.005
Additionally adjusted for z- score of BMI	1 (Ref.)	3.35 (1.71–6.53)	< 0.005

^†^Adjusted for sex, race, Kidmed score (low, medium or high), screen time (continuous) and TV watching during meals (< 3 times/month, 1–4 times/week or ≥ 4 times/week)

## Discussion

In this cross-sectional study of 674 preschoolers from the SENDO project, we found that parents who underestimated their child’s weight had 3.35 -fold higher odds (95% CI: 1.71–6.53) of having unhealthy attitudes towards child’s dietary habits. We previously published that parental attitudes towards child’s dietary habits were inversely associated with child’s nutritional adequacy and diet quality [27], but as far as we know, the association between parental perception of child’s weight and parental attitudes towards child’s dietary habits has previously not been reported. Therefore, our findings are of great interest because they may help pediatricians and public health practitioners identify a subgroup of patients who are most likely to have unhealthy attitudes towards their child’s dietary habits.

Pediatricians may play an important role in parental perception of healthy weight and their preferences regarding obesity counseling [28]. Correct perception of child’s weight is thought to make parents more willing to adopt favorable changes in lifestyle [29], but a recently published systematic review [5] concluded that interventions aiming to correct that misperception may be harmful. We agree with the idea that preventing and treating childhood obesity should be a specialist’s main concern regardless of the perception of the parents [5]. However, our results suggest that asking about the parental perception of their child’s weight may be helpful by identifying a potential group at risk. Efforts should then be directed to identify each unhealthy habit that may exist and to help the family correct these habits individually, progressively and gradually.

Sixteen percent of parents in the SENDO project underestimated their child’s weight. This prevalence is much lower than that reported in previous studies [4], especially considering that information was collected with a verbal scale, which is more prone to error than a visual scale [30]. This difference may be attributed to participants’ idiosyncrasies because most participants in the SENDO project have highly educated parents.

Beyond the scale used, several reasons have been proposed to explain why some parents tend to underestimate their child’s weight status. First, they may be reluctant to acknowledge that their child is overweight or obese because of the social stigma attached to it [31]. Second, the increased prevalence of childhood obesity may alter their perspective, so that when they perceived their child’s weight is similar to that of a large part of the population of his/her age, they may incorrectly conclude it is normal[3]. Related to this alteration of perspective, family history of obesity [4, 7] also has been suggested to be associated with a parental misperception of child’s weight. However, our results did not support this hypothesis. Finally, the image that media offers of obese children is often that of extreme cases of morbid obesity, which may lead to a normalization of cases of overweight or slight obesity [4].

We also observed a strong association between child’s BMI *z* score and parental perception of child’s weight. This finding agrees with previous studies which suggested that child’s z score of BMI was the strongest predictor of parental underestimation. Importantly, most of the evidence suggesting a positive association between child’s z score of the BMI and the odds of parental underestimation of child’s weight comes from cross-sectional studies, wherein reverse-causality bias cannot be discharged [6, 32]. Prospective studies assessing the association between parental perception of child’s weight and weight gain are scarce, and these studies are not exempt from limitations, such as suboptimal control of confounding [31, 33] or inconsistency in the definition of the exposure [31, 34]. Other factors that have been suggested to be associated with parental underestimation of child’s weight are sex and age. Our results do not support previous evidence probably because of the low variability in participant’s age and because differences in fat distribution and secondary sexual characters between boys and girls are non-existent at 5 years old, which was the mean age of the participants in our study.

**Fig. 1:**
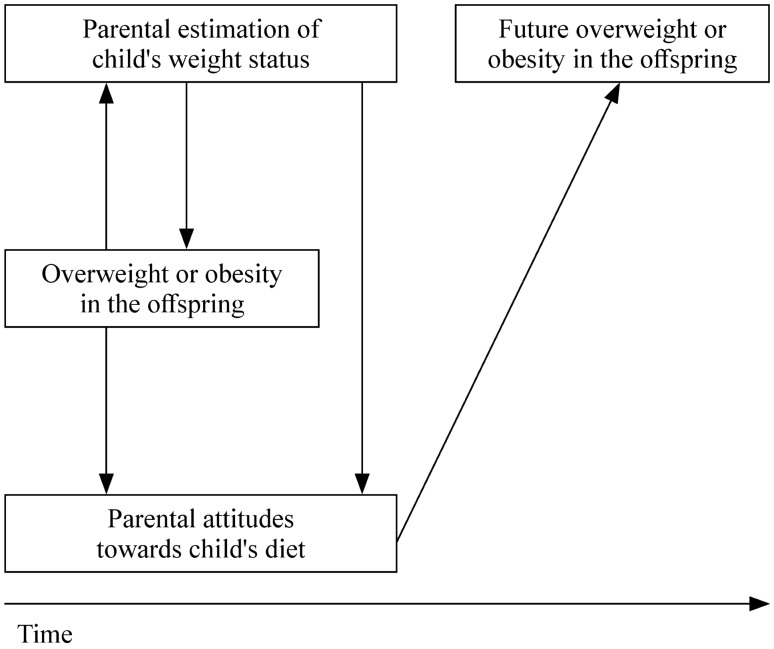
Directed acyclic graph (DAG) of the hypothesized association between parental underestimation of their offspring’s weight status, parental healthy dietary attitudes and offspring’s risk of overweight or obesity

Fully understanding the relation between parental perception of child’s weight and his/her risk of obesity is very complex because the association between parental underestimation of child’s weight and his/her prospective risk of obesity is confounded with child’s actual weight (Fig. 1). In this scenario, our study is of great importance because parental attitudes towards child’s dietary habits could be a mediator in this association. Previous studies showed that dietary habits are associated with weight change [35] and cardiovascular disease [36] in adult populations. Nevertheless, the hypothesis that unhealthy dietary attitudes towards one’s child’s dietary habits lead to a higher risk of the child be overweight or obese needs to be confirmed in prospective studies.

Some limitations of our study must be acknowledged. First, owing to the cross-sectional design of the study, causality cannot be inferred. Cross-sectional studies are susceptible to reverse-causality bias, and therefore we cannot completely reject the association between weight misperception and parental attitudes towards child dietary habits as being in the opposite direction or even bidirectional. Second, we used self-reported information, which is susceptible to misclassification bias. Nevertheless, a validation study of the anthropometric measures reported by parents of participants in the SENDO project showed high correlation coefficients between the measured and the reported data [37]. Third, our sample was not representative of the pediatric population. Participants in the SENDO project are mostly white children with highly educated parents from a developed European country. Although this factor may hamper the generalizability of our results, it also has some benefits, such as higher validity of the self-reported information and reduction of potential confounding by socio-economic variables [38]. However, we acknowledge that the homogeneity of the sample, with little prevalence of overweight or obesity, may have affected our results and may have led to wide confidence intervals. Lastly, although the questionnaire on parental attitudes towards child’s dietary habits has not been validated, previous studies on adult populations showed that it is associated with diet quality, weight change and cardiovascular disease [35, 36, 39].

Despite these limitations, our study has several strengths. Our findings add to the existing evidence by providing data which suggest that parental perception of child’s weight is associated with their attitudes towards child’s dietary habits. Our findings thus suggest a link between parental misperception of child’s weight and his/her future risk of overweight or obesity. In addition, the baseline questionnaire of the SENDO project is extensive and allowed for the control of several confounders.

In conclusion, we found that parental underestimation of child’s weight was associated with 3.35-fold higher odds (95% CI: 1.71 – 6.53) of unhealthy attitudes towards child’s dietary habits, which we hypothesized may be the link between parental misperception and prospective weight gain. Although the relationship between parental perception, child’s actual weight status and dietary attitudes may be complex, we believe that pediatricians and public health practitioners should pay attention to the parental perception of their child’s weight to identify parents who underestimate it as an at-risk group in which to inquire about their lifestyle and dietary habits.

## Supplementary Information

Below is the link to the electronic supplementary material.

Supplementary file1 (DOCX 16 KB)

## Data Availability

The datasets generated during and/or analysed during the current study are available from the corresponding author on reasonable request.
