# Supplementary material for: Parental perception of child's weight, their attitudes towards child's dietary habits and the risk of obesity
Source: World J Pediatr. 2022 Mar 25;18(7):482–9. doi: 10.1007/s12519-022-00540-6 (PMC9205829; doi:10.1007/s12519-022-00540-6)
Supplement: Supplementary file 1 — Supplementary file1 (DOCX 16 KB) [file 12519_2022_540_MOESM1_ESM.docx]

**Supplementary Table I. Multivariable adjusted Odds Ratio (OR) and 95% Confidence Interval (CI) for parental unhealthy attitudes towards child’s dietary habits.**

|  | OR (95% CI) | p |
| --- | --- | --- |
| Parental perception of child’s weight status |  |  |
| Correct estimation | 1.00 (Ref.) |  |
| Underestimation | 3.35 (1.71 – 6.53) | <0.005 |
| Sex (female) | 1.95 (0.96 – 3.93) | 0.06 |
| Race (white) | 0.91 (0.86 – 9,67) | 0.94 |
| Z-score of the BMI | 0.78 (0.54 – 1.11) | 0.17 |
| Kidmed score |  |  |
| Low (≤ 3 points) | 1.00 (Ref.) |  |
| Medium (4-7 points) | 0.29 (0.12 – 0.68) | <0.005 |
| High (≥ 8 points) | 0.19 (0.05 – 0.68) | 0.01 |
| Tv watching during meals |  |  |
| ≤3 times/month | 1.00 (Ref.) |  |
| 1-3 times/week | 1.21 (0.51 – 2.96) | 0.68 |
| ≥4 times/week | 0.90 (0.33 – 2.22) | 0.86 |
| Screen time (hours/day) | 1.20 (0.72 – 1.98) | 0.47 |

Each variable is adjusted for the others in the same model.
